# Acute Pancreatitis as a Complication of Antiepileptic Treatment: Case Series and Review of the Literature

**DOI:** 10.3390/pediatric13010014

**Published:** 2021-02-26

**Authors:** Agnieszka Pawłowska-Kamieniak, Paulina Krawiec, Elżbieta Pac-Kożuchowska

**Affiliations:** Department of Pediatrics and Gastroenterology, Medical University of Lublin, 20-059 Lublin, Poland; paulina.krawiec@umlub.pl (P.K.); elzbietapackozuchowska@umlub.pl (E.P.-K.)

**Keywords:** acute pancreatitis, valproic acid, antiepileptic drugs, acute epigastric pain

## Abstract

Acute pancreatitis (AP) appears to be rare disease in childhood. In children, it has a different aetiology and course, and requires different management than in adult patients. The diagnosis of AP is based on at least two of the three criteria, which include typical clinical symptoms, abnormalities in laboratory tests and/or imaging studies of the pancreas. There are many known causes leading to AP in children including infections, blunt abdominal trauma, genetic factors, gallstone disease, metabolic disorders, anatomical defects of the pancreas, systemic diseases, as well as drugs, including antiepileptic drugs, and especially preparations of valproic acid. In our study, we present four cases of young patients diagnosed with acute pancreatitis as a complication of valproic acid therapy and we present a review of the literature. We believe that the activity of pancreatic enzymes should be monitored in children treated with valproate preparations in the case of clinical symptoms suggesting AP.

## 1. Introduction

Acute pancreatitis (AP) is an acute inflammation resulting from premature activation of pancreatic proenzymes (mainly trypsin) and is associated with various degrees of damage to the pancreas, adjacent tissues and sometimes distant organs [1,2]. The diagnosis of AP is based on at least two of the following three criteria: typical clinical symptoms, an at least three-fold increase over the upper normal limit of pancreatic enzyme activity in the serum and characteristic results of pancreatic imaging (mainly ultrasound or computer tomography (CT), or, less frequently, magnetic resonance imaging (MRI) [2,3,4,5]. AP is more prevalent in adults (5–60 cases per 100,000 persons per year) than in children (3–13 cases per 100,000 persons per year) [6]. Pancreatitis in children has a different aetiology and course, and requires different management than in adult patients [3,6,7].

There are many known causes leading to AP in children including infections, blunt abdominal trauma, genetic factors, gallstone disease, metabolic disorders, anatomical defects of the pancreas, systemic diseases, as well as drugs, including antiepileptic drugs [1,2,3,6,8,9].

Characteristic symptoms of AP include epigastric pain, persistent nausea and vomiting [8,10]. 

In children treated with antiepileptic drugs, the diagnosis of pancreatitis should be taken into consideration in the case of clinical symptoms suggesting AP. The early diagnosis of acute pancreatitis allows for implementing appropriate therapy and monitoring the course of pancreatitis [4,5,7,8,10,11,12,13]. After the diagnosis of AP, fluid resuscitation is recommended to correct hypovolemia and to help preserve adequate perfusion in the pancreas. In the past, patients with AP were managed by following diet zero and being provided with parenteral nutrition. It was believed that the presence of food in the intestines would stimulate cholecystokinin (CCK) release, which in turn would stimulate pancreatic enzyme secretion, which could lead to further activation of proteolytic enzymes and increased pancreas injury. Currently, since 2018, as agreed by North American Society for Pediatric Gastroenterology, Hepatology and Nutrition (NASPGHAN), it has been recommended that children with mild acute pancreatitis may benefit from early (within 48–72 h of presentation) oral or enteral nutrition to decrease the length of stay and decrease the risk of organ dysfunction. Painkillers should be included in the treatment of acute pancreatitis as well [7,14].

In our study, we present four cases of patients diagnosed with AP as a complication of valproic acid therapy of epileptic paroxysmal disorders.

## 2. Case Reports

### 2.1. Patient 1

A 7-year-old boy treated for two months with valproic acid because of epilepsy was admitted to the Department of Paediatrics and Gastroenterology, Medical University in Lublin for epigastric pain and vomiting. On admission, the serum amylase activity was 982 U/L (reference value up to 118 U/L) and lipase activity of 1523 U/L (reference value up to 51 U/L) was found. The abdominal ultrasound showed a slightly swollen pancreas with decreased echogenicity. The serum valproate level was within therapeutic limits. 

### 2.2. Patient 2 

A 4-year-old girl with Smith–Lemli–Opitz syndrome was admitted to the Department of Paediatrics and Gastroenterology, Medical University in Lublin due to abdominal pain. She was treated with valproate due to epilepsy. The dose of drug had been increased two weeks earlier to achieve better control of seizure events. On admission, the serum amylase activity was 234 U/L and the lipase activity was 495 U/L. Abdominal ultrasonography did not reveal any abnormalities. The serum valproate level was within therapeutic range. According to the neurologist’s recommendation, valproic acid was switched to levetiracetam. However, due to the lack of a sufficient antiepileptic effect of this therapy, after six months of first AP episode in our patient, valproic acid was reintroduced. After two months, the girl was admitted to our department again because of abdominal pain. Highly increased activity in the serum amylase (1936 U/L) and lipase (2433 U/L) was stated. The pancreas image in the ultrasound examination was unchanged.

### 2.3. Patient 3

A 15-year-old boy treated with valproic acid for several years due to epilepsy was admitted to the Department of Paediatrics and Gastroenterology, Medical University in Lublin because of abdominal pain. Two weeks before admission to our department, the dose of the antiepileptic drug had been increased to improve seizure control. On admission, the activity of the serum amylase was 562 U/L, and lipase, 1362 U/L. The initial abdominal ultrasound examination did not reveal any abnormalities. The level of valproic acid in the serum was within the therapeutic range. Although the typical treatment (diet zero, painkillers for the first few days) was introduced, the patient’s condition did not improve. The laboratory results showed an increase in inflammatory markers and the progressive increase in pancreatic enzymes in the serum. The computer tomography (CT) scans performed on the fourth day of hospitalization revealed two foci of necrosis in the pancreas. Intensive treatment was applied, including broad-spectrum antibiotic (meropenem) and total parenteral nutrition achieving the resolution of AP symptoms within one week. In the fourth week of hospitalization, CT scans showed a normal image of the pancreas and the laboratory results revealed normal activity of pancreatic enzymes.

### 2.4. Patient 4

A 17-year-old boy with epilepsy was admitted to the Department of Paediatrics and Gastroenterology, Medical University of Lublin because of abdominal pain. For two years, the patient had several modifications of antiepileptic therapy due to insufficient efficacy and/or poor tolerability of the drug. Two months before admission to our department, the boy started valproic acid therapy. In the laboratory results, amylase activity was 232 U/L and lipase activity was 534 U/L. The abdominal ultrasound examination showed heterogeneous echogenicity of the pancreas. 

In all patients, the typical treatment of acute pancreatitis was applied, previous antiepileptic treatment was changed to levetiracetam or anti-epileptic drugs instead of valproate, achieving the resolution of symptoms and the normalization of pancreatic enzymes. Except for patient 3, who was re-treated with valproic acid, no relapse of acute pancreatitis was observed in the patients described.

## 3. Discussion

Acute pancreatitis is less frequent in children than in adult patients. However, it should be always suspected in patients with epigastric pain and accompanying nausea or vomiting. The diagnosis of acute pancreatitis is based on at least two of the three criteria, which include typical clinical symptoms and abnormalities in laboratory tests and/or imaging studies of pancreas [2,3,4,5,6,7,8,11].

A characteristic symptom of AP is abdominal pain in the epigastric area, which occurs suddenly and usually has a continuous and dull character. In children with AP, abdominal pain rarely radiates to the back, in contrast to adult patients with AP. Abdominal pain may be accompanied by nausea or vomiting. According to the diagnostic criteria, the increase in the activity of pancreatic enzymes in the serum at least three times over the upper normal limit is characteristic for AP. The activity of amylase in AP increases earlier than the serum lipase activity. However, lipase activity remains elevated longer than amylase. The sensitivity of lipase in AP is higher (96.6%) than amylase (78.6%). However, the specificity of both enzymes is comparable, and is 99.1% for lipase and 99.4% for amylase [4,7,8,10,13,15,16,17,18].

There are three forms of acute pancreatitis. The first one—mild—is featured as interstitial inflammation, with a minor dysfunction of the organ. It is a self-limiting process, which occurs mostly in children. The moderate form of AP is characterized by local complications and temporary impairment of organ function. The severe form of AP is rare in children and is associated with organ dysfunction lasting for more than 48 h. The acute necrotizing pancreatitis, which occurs very rarely in children, is associated with the activation of many mediators, as well as proinflammatory cytokines. As a result of the imbalance between the inflammatory process and the compensatory anti-inflammatory response, the local inflammation may change into systemic inflammation complicated by organ failure. It occurs less frequently in children than in adults with AP [1,4,5].

There are many aetiological factors of acute pancreatitis. Infections, particularly viral, are one of the most common causes of AP. They may lead to direct damage to the pancreatic parenchyma. Blunt abdominal trauma, during which pancreatic enzymes are released from damaged cells, is also a well-established aetiological factor of AP in paediatric patients. Genetic factors including mutations in CFTR, PRSS1, SPINK1 genes play an important role among the causes of acute, recurrent pancreatitis [1,2,3,6,8].

Other aetiological factors of acute pancreatitis in children are cholelithiasis, Oddi’s sphincter dysfunction and metabolic disorders, including hypercalcemia and hyperlipidaemia [3,8]. Less frequently described causes are anatomical defects of the pancreas or parasitic infestation. Acute pancreatitis may occur in the course of systemic diseases such as systemic lupus erythematosus and Schönlein–Henoch’s disease. Rarely, the acute pancreatitis occurs in the course of inflammatory bowel diseases (IBDs), as a result of oedema and fibrosis of the bile ducts or as a complication of azathioprine treatment [2,3,6,8].

The onset of the inflammatory process in the pancreas may be also induced by drugs such as salicylates, acetaminophen, steroids, cytostatic drugs, immunosuppressive drugs, tetracyclines, erythromycin, and antiepileptic drugs [8,10,12,16,18]. It is believed that pancreatitis as a drug-induced acute pancreatic inflammation is thought to account for 0.1%–2% of all AP. The majority of this form of pancreatitis is mild to moderate in severity [4,5,18]. 

Valproic acid preparations used in anticonvulsant therapy are quite a common cause of hepatotoxicity and may also lead to acute pancreatitis. Trivedi CD et al. classified valproate as the first group drug, causing pancreatitis more often than other drugs [19]. Likewise, valproic acid is classified by Badalov N. et al. [16]. According to the WHO, valproic acid is one of the known and recognized substances associated with drug-induced pancreatitis [8]. Mostly, AP resulting from valproate therapy is observed in the paediatric population. This is related to the more frequent use of these drugs in younger patients to control seizures [1,8,9,13,18]. The mechanism of valproate toxicity is not fully known. Most likely, it may have the character of idiosyncrasy, which is confirmed by the fact of the correct therapeutic concentration of valproate in the serum of patients with AP [1,4,5,8,9,10,16]. Other theories consider the increase in free radicals and endothelial permeability under the influence of the metabolic products of valproic acid in the liver [4,8].

Acute pancreatitis associated with valproic acid therapy was described for the first time in 1979 [1,8,10,11].

Although among drug-induced pancreatitis, valproate derivatives are often mentioned as the cause of acute pancreatitis, among all patients treated with valproate, AP complications are not common.

Pellock JM. et al., on the basis of 34 clinical trial analyses, found that AP is a rare complication of valproic acid therapy. However, in the case of symptoms suggesting pancreatitis and increased activity in pancreatic enzymes, antiepileptic therapy with valproic acid should be discontinued due to possible individual susceptibility of the pancreas to discontinued treatment [10].

Ali A. et al. described the case of a 21-year-old patient with mental retardation and epilepsy in whom necrosis of the pancreatic body and tail was diagnosed during laparotomy. The patient was treated with valproate since early childhood. The dose of the antiepileptic drug was increased due to improved seizure control. Two weeks later, the patient was admitted to the hospital with acute abdominal symptoms. The controlled level of valproate in the blood serum did not exceed the therapeutic values [1].

In a retrospective study, Bai HX. et al. analyzed drug-induced pancreatitis among patients hospitalized at Yale–New Haven Children’s Hospital from 1994 to 2007, comparing the course of disease to pancreatitis of distinct aetiology. It was found that the most common cause of drug-induced pancreatitis in the study group was the use of antiepileptic drugs and those used in the therapy of acute lymphatic leukaemia (ALL), in 20% of cases, respectively. The highest proportion of valproate in the aetiology of acute pancreatitis was found in patients under 10 years of age [8].

Guevara-Campos J. et al. described a 7-year-old girl with epilepsy who was treated with valproic acid and developed epigastric pain. Based on a physical examination, laboratory study, ultrasound and computer tomography, acute pancreatitis was recognized. The level of valproate in the blood serum was in therapeutic values [11].

Cofini M. et al. reported four cases of acute pancreatitis induced by valproic acid in a group of 52 cases, all of acute pancreatitis in children treated from January 2008 to December 2012 [9].

Quan W. et al. described the case of a 26-year-old patient treated for mental disorders with valproic acid. After less than a week of treatment, the patient developed abdominal pain, flatulence and vomiting. Based on the diagnostic process, acute pancreatitis was recognized. Due to this complication of the therapy, the treatment of the main disease was changed [12].

Meczker A. et al., in a study in May 2019 analyzing PubMed, EMBASE and the Cochrane Library, found 1060 eligible cases in 856 reports. In their analysis, drug-induced acute pancreatitis caused by anticonvulsants was found in 103 cases (9.72%) [18]. 

Trivedi CD. et al., based on the data from the National Library of Medicine/Pubmed, reported cases of drug-induced pancreatitis from 1966 to 30 April 2004. They reported 80 cases of AP that was related with valproate. Valproic acid, among more than one hundred medications, was classified as class I drug, most often associated with complications such as acute pancreatitis [19].

Badalov N. et al. also analyzed data from the Medline database throughout 1955–2006. They analyzed 1214 case reports that allowed for the classification of 120 drugs connected with drug-induced pancreatitis. Among all the cases analyzed, 35 were related to valproate and three quarters of these cases occurred in pediatric patients [16]. 

Vinklerova I. et al. conducted a retrospective analysis of all cases of pancreatitis in the University Hospital in Olomouc from 2006–2007. Among 170 of all AP cases analyzed by researchers, 5.3% represented drug-induced acute pancreatitis, and among them, 11.2% were non-biliary cases [15]. 

Randall MM. et al. reported cases of all patients below 13 years of age between 2006 and 2016 who presented to the pediatric emergency department at Loma Linda University Medical Center with a final diagnosis of pancreatitis. Researchers revealed in their work that sixteen patients (19%) of acute pancreatitis cases were drug-induced and valproic acid was in the first position among the applied drugs [17].

Jain A. et al. reported the case of a 22-year-old man with generalized tonic–clonic seizures, who developed acute pancreatitis after being treated with valproate for 6 months [5]. 

Grauso-Eby NL. et al. presented in their work four children with pancreatitis induced by valproic acid. Also based on Medline and PubMed within a 4-year period (1995–1999) they found 33 cases of drug-induced AP and among them there were the described patients. The researchers recommended that clinicians maintain a high index of suspicion for pancreatitis in any child taking valproic acid who present with nonspecific abdominal symptoms [13]. 

In the four patients described in this paper, the main aetiological factor of acute pancreatitis was the valproic acid used to control epileptic seizures. In two patients, typical symptoms of pancreatitis appeared shortly after increasing the dose of the drug. In two other patients, symptoms associated with pancreatitis appeared shortly after the introduction of valproic acid therapy.

In each of our patients with drug-induced acute pancreatitis, the level of valproic acid was within normal ranges, which may indicate a mechanism of idiosyncrasy underlying the toxic action of this drug.

## 4. Conclusions

The activity of pancreatic enzymes should be monitored in children treated with antiepileptic drugs, including valproate preparations, particularly in the case of clinical symptoms suggesting acute pancreatitis.

## Data Availability

Not applicable.
